# Biologic Data of Cynomolgus Monkeys Maintained under Laboratory Conditions

**DOI:** 10.1371/journal.pone.0157003

**Published:** 2016-06-09

**Authors:** Marilena Caterina Rosso, Paola Badino, Giulio Ferrero, Roberto Costa, Francesca Cordero, Stephanie Steidler

**Affiliations:** 1 Department of Veterinary Science, University of Turin, Grugliasco (TO), Italy; 2 RBM SpA - Merck Serono, Ivrea, Italy; 3 Department of Computer Science, University of Turin, Turin, Italy; 4 Department of Clinical and Biological Sciences, University of Turin, Orbassano, Turin, Italy; 5 Center for Molecular Systems Biology, University of Turin, Orbassano, Turin, Italy; CEA, FRANCE

## Abstract

The cynomolgus monkey (*Macaca fascicularis*) is a well-known non-human primate species commonly used in non-clinical research. It is important to know basal clinical pathology parameters in order to have a reference for evaluating any potential treatment-induced effects, maintaining health status among animals and, if needed, evaluating correct substantiative therapies. In this study, data from 238 untreated cynomolgus monkeys (119 males and 119 females of juvenile age, 2.5 to 3.5 years) kept under laboratory conditions were used to build up a reference database of clinical pathology parameters. Twenty-two hematology markers, 24 clinical chemistry markers and two blood coagulation parameters were analyzed. Gender-related differences were evaluated using statistical analyses. To assess the possible effects of stress induced by housing or handling involved in treatment procedures, 78 animals (35 males and 35 females out of 238 juvenile monkeys and four adult males and four adult females) were used to evaluate cortisol, corticosterone and behavioral assessment over time. Data were analyzed using a non-parametric statistical test and machine learning approaches. Reference clinical pathology data obtained from untreated animals may be extremely useful for investigators employing cynomolgus monkeys as a test system for non-clinical safety studies.

## Introduction

Non-human Primates (NHP) are the closest phylogenic relatives to man which makes them a suitable and sometimes indispensable model in many fields of biomedical research [1–4]. *Macaca fascicularis* (commonly called cynomolgus monkey) is a well-known NHP species widely used in various types of studies in pharmaceutical (toxicological, pharmacological, metabolic, physiological and psychological) research and development [5–9]. At present, the use of animals in biomedical research is limited to pre-screening tests, initial drug development phases, or when there is the supplementary aim of giving indications of the species to use in research projects on animals [10–12]. Nevertheless, issues may be raised regarding their management and particularly the ethics of their use, over and above the intrinsic issues of testing on other animal species [13–15]. Historical colony data and reference ranges of hematology and clinical chemistry parameters are valuable complements to scientific investigations [16] as they are essential in evaluating the effects induced in laboratory animals by molecules during experimental studies [6, 7, 17–19]. Baseline biological databases are also important to evaluate the health status of animals prior to their use for scientific purposes and to manage diagnosis as well as to improve therapies and experimental techniques [7, 8].

Evaluation of laboratory data should take into account possible differences in baseline values due to different animal sources and to the different analytical methods used in individual laboratories [6–9, 19]. Physiological responses to stress conditions and induced reactions based on husbandry practice may also induce changes in clinical pathology parameters. Over time, with adequate training and habituation, variability and extent of response may decrease [20, 21].

A number of parameters, namely cortisol, neutrophil/leukocyte (N/L) ratio, and white blood cells are known to vary due to stress-inducing conditions. In particular, circulating cortisol levels are known to increase in response to various kinds of stress in different animal species including NHP [22–26].

The aim of this study was to establish an internal database of physiological and clinical pathology parameters, collected from *Macaca fascicularis* during the course of toxicology studies. Several statistical and machine learning approaches were applied to these data to achieve a panel of parameters suitable to assess animal welfare and to identify potential relations between stress, age, housing and treatment methods in *Macaca fascicularis*.

## Materials and Methods

### Cynomolgus monkeys (*Macaca fascicularis*)

The 246 cynomolgus monkeys (123 males and 123 females) used were of Chinese origin, provided by RC Hartelust BV, Tilburg (The Netherlands). At the start of the investigation, the animals were about 2.5 to 7.5 years old with a body weight in the range approximately from 2.5 to 7.5 kg. Prior to their arrival at the Test Facility, all monkeys had been quarantined upon arrival in Europe according to ruling EEC (European Economic Community) regulations and identified with a number tattooed by the supplier. All animals had tested negative for tuberculosis, Herpes B, Simian Immunodeficiency Virus, Simian T-cell Leukemia Virus, Simian Retrovirus, Marburg and Ebola-like viruses and were dewormed. Additional microbiological and parasitological examinations of feces and clinical examination were carried out in all animals upon their arrival at the premises. Animal data used in this study derived from animals inserted in specific Non clinical Safety protocols (timeframe 2013–2015) conducted according to the existing four international guidelines: (i) ICH Guideline M3 (Nonclinical Safety Studies for the Conduct of Human Clinical Trials and marketing authorization for Pharmaceuticals), (ii) ICH Guideline S6 (Preclinical Safety Evaluation of Biotechnology-Derived Pharmaceuticals), (iii) ICH Guideline S7a (Safety Pharmacology Studies for Human Pharmaceuticals), and (iv) ICH Guideline S9 (Nonclinical Evaluation for Anticancer Pharmaceuticals). Protocols were approved internally by the animal science and welfare officer and by the person responsible for the use of animals for experimental purposes. The site is fully authorized by the Italian Ministry of Health to perform these studies. The study described was carried out at RBM S.p.A.—Istituto di Ricerche Biomediche *A. Marxer*—Via Ribes, 1–10010 Colleretto Giacosa (TO), Italy. The overall project described in this paper was approved by the test site management and animal welfare officer.

### Housing and environment conditions

Monkeys were housed in stainless steel cages, with front and bottom in stainless steel grill. Animal housing meets Italian requirements for laboratory animal welfare. The monkeys were housed singly or two or three animals per cage. Environmental enrichment was offered to animals in the form of alimentary and tactile enrichment as well as toys such as hanging balls, mirrors and internal manipulanda. The protection of animals used, housing, and welfare are in accordance with Italian law No. 26 March 4th, 2014 and Directive 2010/63/EU. However, at the time when this study was conducted, some animals were housed singly (if means and justifications were available during toxicology study protocols) in compliance with Italian Law No. 116 January 27th, 1992 adopting Directive 86/609/EEC on the Protection of Animals used for Experimental and other Scientific Purposes. Animal facilities at the test site are inspected at least once every year by the Ministry of Health. Animals were housed in an air conditioned room in the following ambient conditions: 22°C ± 2°C temperature, 15–20 air changes per hour, relative humidity 55% ± 15 and artificial lighting with a circadian cycle of 12 hours from 7 a.m. to 7 p.m.

### Diet and water supply

A diet coded *KE25 GLP top certificate*, produced by Mucedola S.r.l., Settimo Milanese (Italy) and analyzed for nutrients was provided. The diet was distributed once a day, in the total amount of about 200 g/monkey. Dry and fresh fruit or vegetables were distributed additionally every day. The drinking water offered to the animals *ad libitum* came from the municipal water main and was distributed by means of an automatic watering valve system. Water and diet are periodically analyzed for microbiological count, for the presence of heavy metals, other contaminants (e.g. solvents, pesticides) and other physical and chemical properties on behalf of the Facility. No contaminants that might interfere with the objectives of this study were present either in the diet or in the drinking water.

### Blood and urine sampling

Blood samples were drawn in the morning, prior to performing any treatment or any technical operations, from a peripheral vein of each overnight fasted and conscious (non-anesthetized) monkeys as per the project authorization granted according to the above mentioned Italian Laws No. 116 and 26. Blood samples were collected into test tubes for hematology, coagulation and clinical chemistry tests (0.50 mL into K2EDTA-anticoagulant-treated tubes, 0.9 mL into 3.2% NaCitrate-anticoagulant-treated tubes and 1.1 mL into Serum Separate Tube (SST) tubes, respectively). NaCitrate samples were centrifuged at 1600 x g for 15 minutes and plasma was transferred to a secondary analysis tube. Blood collected in SST tubes was allowed to clot for about 30–60 minutes at room temperature, and then centrifuged at 2200 x g for 10 minutes and serum transferred to a secondary analysis tube. Urine samples were collected from the animals kept in metabolic cages for about 16 hours without food and water, after having received 20 mL/kg of tap water by gavage as water load, the evening prior to scheduled analysis.

All the above procedures were performed by the same technical staff to avoid variations in handling and bleeding. The volume of blood collected was well within guidelines for the species and individual bodyweight [27].

### Clinical pathology analysis

For each parameter, the abbreviations and units are reported in brackets.

*Hematological analysis* was performed using a Siemens ADVIA 2120 instrument. The following parameters were analyzed: erythrocytes (RBC, 10^6^/*μ*L), hemoglobin (HGB, g/dL), hematocrit (HCT, %), mean corpuscular volume (MCV, fL), mean corpuscular hemoglobin (MCH, pg), mean corpuscular hemoglobin concentration (MCHC, g/dL), reticulocytes (RET, % and 10^9^/L), leukocytes (WBC, 10^3^/*μ*L), neutrophils (NEU, % and 10^3^/*μ*L), lymphocytes (LYMPH, % and 10^3^/*μ*L), monocytes (MONO, % and 10^3^/*μ*L), eosinophils (EOS, % and 10^3^/*μ*L), basophils (BAS, % and 10^3^/*μ*L), platelets (PLT, 10^3^/*μ*L), large unstained cells (LUC, %).

*Coagulation analysis* was performed using an ACL7000 instrument, with dedicated reagents. The following parameters were analyzed: prothrombin time (PT, sec), and activated partial thromboplastin time (APTT, sec).

*Lymphocyte immunophenotyping in whole blood* The following lymphocyte subpopulations were determined using a FACSCanto flow cytometer and FACSDiva software: total T cells (CD3^+^), Helper T cells (CD3^+^CD4^+^), Cytotoxic T cells (CD3^+^CD8^+^), Total B cells (CD3^−^CD20^+^), and NK cells (CD3^−^CD16^+^).

*Clinical chemistry analysis* was performed using an Olympus AU400 instrument with dedicated reagents (Beckman Coulter, except for GLDH reagent, supplied by Diasys). The following parameters were analyzed: glucose (GLU, mmol/L), total cholesterol (CHOL, mmol/L), triglycerides (TRIG, mmol/L), urea (UREA, mmol/L), creatinine (CREA, *μ*mol/L), aspartate aminotransferase (AST, U/L), alanine aminotransferase (ALT, U/L), alkaline phosphatase (AP, U/L), gamma glutamyl transferase (GGT, U/L), glutamate dehydrogenase (GLDH, U/L), total bilirubin (TBIL, *μ*mol/L), total protein (TP, g/L), C-reactive protein (CRP, mg/L), sodium (NA, mmol/L), potassium (K, mmol/L), chloride (CL, mmol/L), calcium (CA, mmol/L) and inorganic phosphorus (IP, mmol/L).

*Serum protein electrophoresis analysis* was performed using a Semi-Automated Systems (SAS) / Platinum instrument (Helena Laboratories). The following parameters were analyzed: albumin (ALB, %), alpha1 globulin (%), alpha2 globulin (%), beta globulin (%), gamma globulin (%) and albumin /globulin ratio (A/G ratio, calculated by the SAS system).

*Cortisol* (pg/mL) and *corticosterone* (pg/mL) blood levels were measured using an Enzyme Immunoassay kit (Arbor assays).

*Urine analysis* was performed using an Aution Max instrument, with Uriflet 9 UB strips (Menarini). The following parameters were analyzed: diuresis (mL/16h), specific gravity (SG), pH value (PH), color (COL), turbidity (TURB), glucose (GLUU, mg/dL), protein (PRO, mg/dL), bilirubin (BIL, mg/dL), urobilinogen (URO, mg/dL), blood (BLO, mg/dL), ketones (KET, mg/dL) and sediment (microscopic urine sediment of centrifuged urine samples was examined to confirm erythrocyte presence if hemoglobin was found by instrument analysis).

### Clinical and behavior observations

*Body Weight* (kg): animals were weighed on days not scheduled for blood sampling using an electronic scale (Sartorius CPA 16001S) external to the cage. The behavior of animals was assessed by a *Functional Observational Battery* (FOB) [28]. The following parameters of the FOB were evaluated: number of perchings (number observed during the observation period), lethargy/arousal, posture, salivation, tremor activity, convulsive activity, movement of facial muscles, eyelid closure, lacrimation, piloerection, gait, gait score, bizarre behavior, spontaneous vocalization (presence or absence), pupil response (presence or absence of pupillary light reflex) and approach response. The parameters were either scored according to a scoring system and were evaluated in conscious animals in their home cages.

### Administration routes

Intravenous infusion (IV): prior to infusion, the skin was clipped free from hair. Appropriate disposable, plastic material and infusion pumps were used for administration. A catheter was inserted into a peripheral vein on conscious restrained animals. The catheter was connected to an infusion pump. During infusion (approximately 1.5 hours), the solutions were kept in adequate reservoirs connected to the pump. The volume of solution administered to each animal was adjusted according to the most recent body weight at a rate of 10 mL/kg/h.

Subcutaneous administration (SC): was performed in the subcutis of the thighs, with appropriate disposable sterile syringes and needles and also in this case, before treatment the skin was clipped free of hair. Monkeys received saline solution at the selected dose with a fixed maximum volume of 2 mL/kg, alternately in the right and left thigh. The volume of solution was adjusted according to the most recent individual body weight.

### Data analysis

Biochemical and hematological data were statistically analyzed using the Wilcoxon Rank-Sum test. Results having a p-value < 0.05 were taken into account.

The contribution of each biochemical and hematological measurement to the classification of animal with respect to age, type of administration, and housing was evaluated using Weka 3.6.12 [29]. The machine-learning classification methodology implemented in Weka was based on a logistic classifier with ten fold cross-validation. The contribution of each covariate to the classification results was evaluated using Weka *ChiSquareAttributeEval*. This methodology is based on the independence of the occurrence of a specific attribute and the occurrence of a specific class identity of the animal. It is considered as features all biochemical and hematological measurements and the class identity as the study of a sample belonging to that class (studies defined in Cohort 2). We consider only the features having a Chi-square test score higher than 3.84 that means a significance value (p-value) lower than 0.05 considering a degree of freedom equal to 1. The Chi-square values are converted in p-values.

## Results

The study was aimed at establishing an internal database of physiological and biological parameters of baseline cynomolgus monkey (Macaca fascicularis), a well-known non-human primate species commonly used in non-clinical research. Several statistical and machine learning approaches were applied to this database to achieve a panel of potential parameters suited to assessing animal welfare and to identify potential relations between age, housing and treatment methods in *Macaca fascicularis*. In a first instance, the effect of sex on hematological and clinical chemistry parameters was assessed on a wide number of subjects by means of statistical analysis. Selected subgroups of subjects were then evaluated by a time-course protocol, in order to identify critical measurements through a comparison by age, administration route or housing. In this protocol, circulating levels of cortisol were measured at all time points.

### Protocol design

The study was divided into two cohorts.

**Fig 1:**
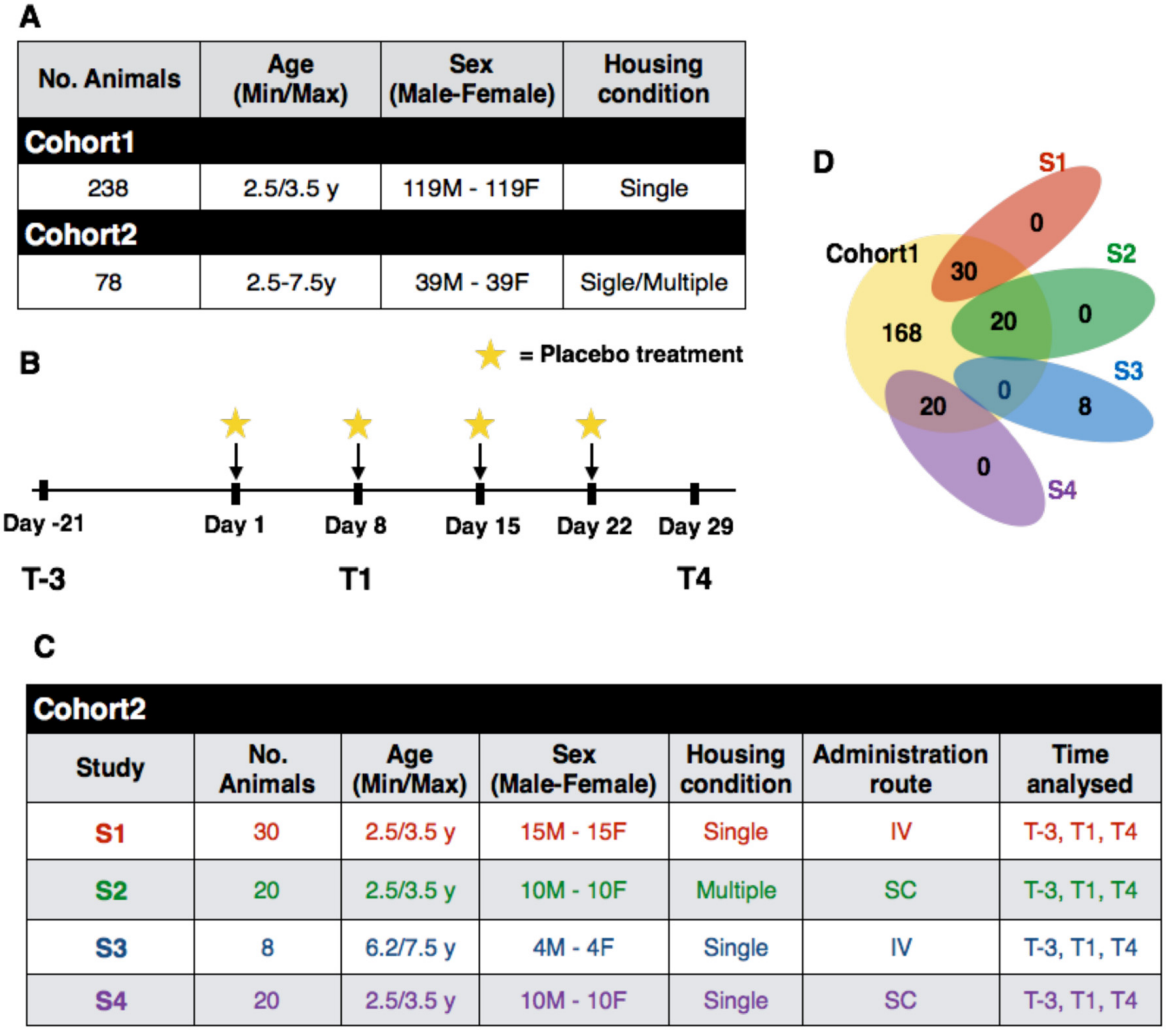
Description of the studies. Panel A reports the features of **Cohort 1** and **Cohort 2** in terms of number of animals, age, sex and housing condition. Panel B reports the features of animals in **Cohort 2**, grouped by the study to which they belong. Panel C shows a Venn diagram showing overlapping of animals in **Cohort 1** and **Cohort 2**. Panel D summarizes the experimental design used to collect biological samples at different time points. The star symbol represents the placebo treatment performed. Note that on Day 8 before the intravenous or subcutaneous administration of the physiological solution, was collected the blood and urine samples. S = Study, IV = IntraVenous infusion, SC = SubCutaneous injection.

The **Cohort 1** composed of 238 animals having an age ranging between 2.5 and 3.5 years, whose features are reported in the first row of Fig 1A. The **Cohort 1** was used to measure the effects of sex on hematological and biochemical measurements, all hematological and biochemical measurements are reported in S1 Table.

The **Cohort 2** composed of 78 animals (39 males and 39 females) whose features are summarized in the second row of Fig 1A. This cohort was used to compare the hematological, biochemical, hormonal and behavioral parameters considering the animals’ age, housing and administration route in a time-course protocol, in which, in addition to hematological and biochemical parameters, cortisol and corticosterone serum levels and FOB were also measured. The protocol, applied to **Cohort 2**, consisted of repeated intravenous or subcutaneous administration of the control item (physiological solution) once a week for four consecutive weeks. In particular, the evaluation were carried out on the 78 monkeys three weeks prior to the first administration (T-3), and then one week after the first administration (T1), and one week after the fourth administration (T4) of saline solution. Fig 1B outlines the experimental design used to collect blood samples at different time points. The protocol was applied on all 78 animals that are split in four different studies (S1–S4). Each study has associated to three features: age of animal (juvenile or older), housing condition (single or multiple) and administration route of saline solution (intravenous infusion or subcutaneous administration). Details about all studies are reported in Fig 1C. All data about the four studies are reported in the S2 Table.

The overlapping of animals in **Cohort 1** and the four studies of **Cohort 2** is highlighted in Fig 1D.

### Effects of gender on hematological and biochemical measurements

**Fig 2:**
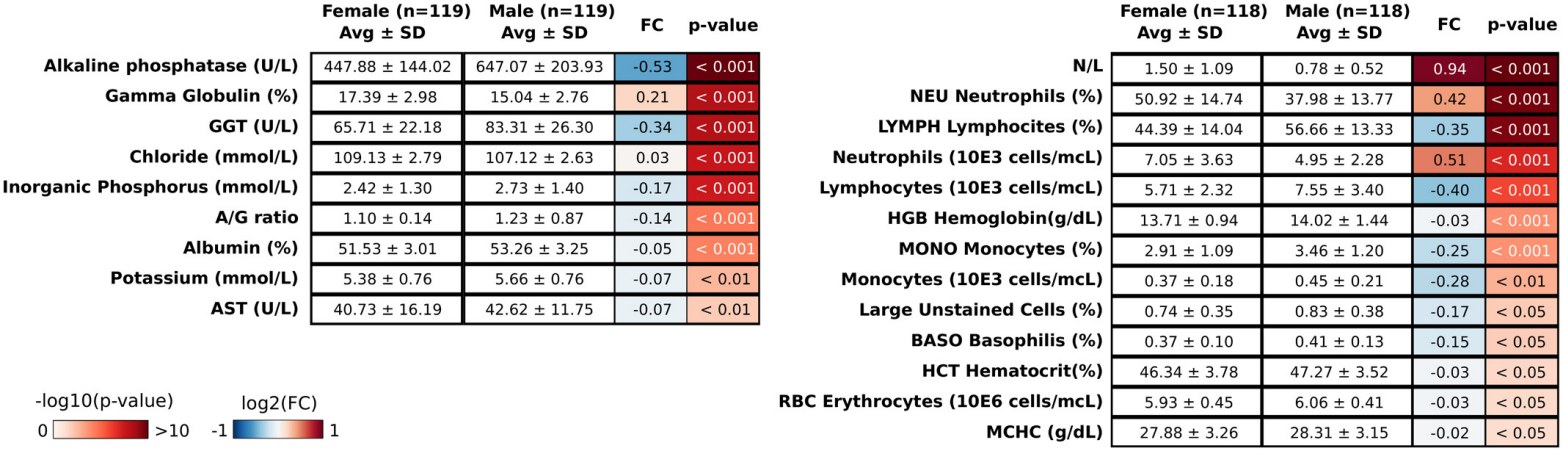
Set of hematological and biochemical measurements significantly different between female and male in Cohort 1. Each measurement corresponds to four columns: the average value and the standard deviation for females (i) and males (ii), (iii) log2 Fold Change (FC) of the measurements computed as females versus males (both represented as heat maps), and (iv) the p-values computed by Wilcoxon Rank-Sum test.

The effect of gender on hematological and biochemical measurements was evaluated on the **Cohort 1**. The mean body weight measured in our animals was 3.1 Kg in males and 2.8 Kg in females. The difference was statistically significant. Differences in hematological and biochemical measurements between males and females were statistically evaluated by the Wilcoxon Rank-Sum test. In Fig 2 we set out the 22 measurements associated to a p-value < 0.05 and associated fold change computed between female and male mean values of the significant hematological and biochemical measurements. A S1 Fig shows the p-values and fold change values of all the 48 measurements.

### Identification of critical measurements

The cynomolgus monkeys involved in the **Cohort 2** (n = 78, 39 males and 39 females) were included in the time-course protocol explained above. In addition to hematological and biochemical parameters, cortisol and corticosterone serum levels were measured as well as FOB and urine analysis.

The aim was to identify hematological and biochemical measurements common to the animals considering their **age** by comparing young animals (S1+S2+S4) to adults (S3), **housing** by comparing young animals given the control item by subcutaneous administration and kept in multiple cages (S2) versus those in single cages (S4), and **administration route** comparing young animals in single cages given subcutaneous injections (S4) versus intravenous infusion (S1). In these analyses, each time point was considered individually.

At time T-3 animals were divided into two groups, the first consisting of S1, S2, and S4 and the second of S3 since the only difference between them was due to age. After the first IV or SC administration, the animals were housed singly or 2/3 per cage.

Body weight was evaluated separately in juvenile and adult animals in view of the plausible and physiological, statistically significant difference, with the adults’ weight being in both sexes remarkably higher than that of the young ones.

Not all parameters were measured at each time point. For this reason all Figures report the number of samples used. Moreover, S3 Table reports the number of animals measured for each parameter, time point and study.

Before conducting statistical analysis among the studies, we tested the gender effect on cortisol and corticosterone at time T-3. Cortisol was measured in 19 males and 19 females, and the difference was statistically evaluated by the Wilcoxon Rank-Sum test obtaining a p-value equal to 0.58. The cortisol level was higher in males, the fold change value being equal to 0.66. Corticosterone was measured in the same number of animals, the p-value obtained being < 0.001. The corticosterone level was higher in males, with a fold change equal to 0.75.

**Fig 3:**
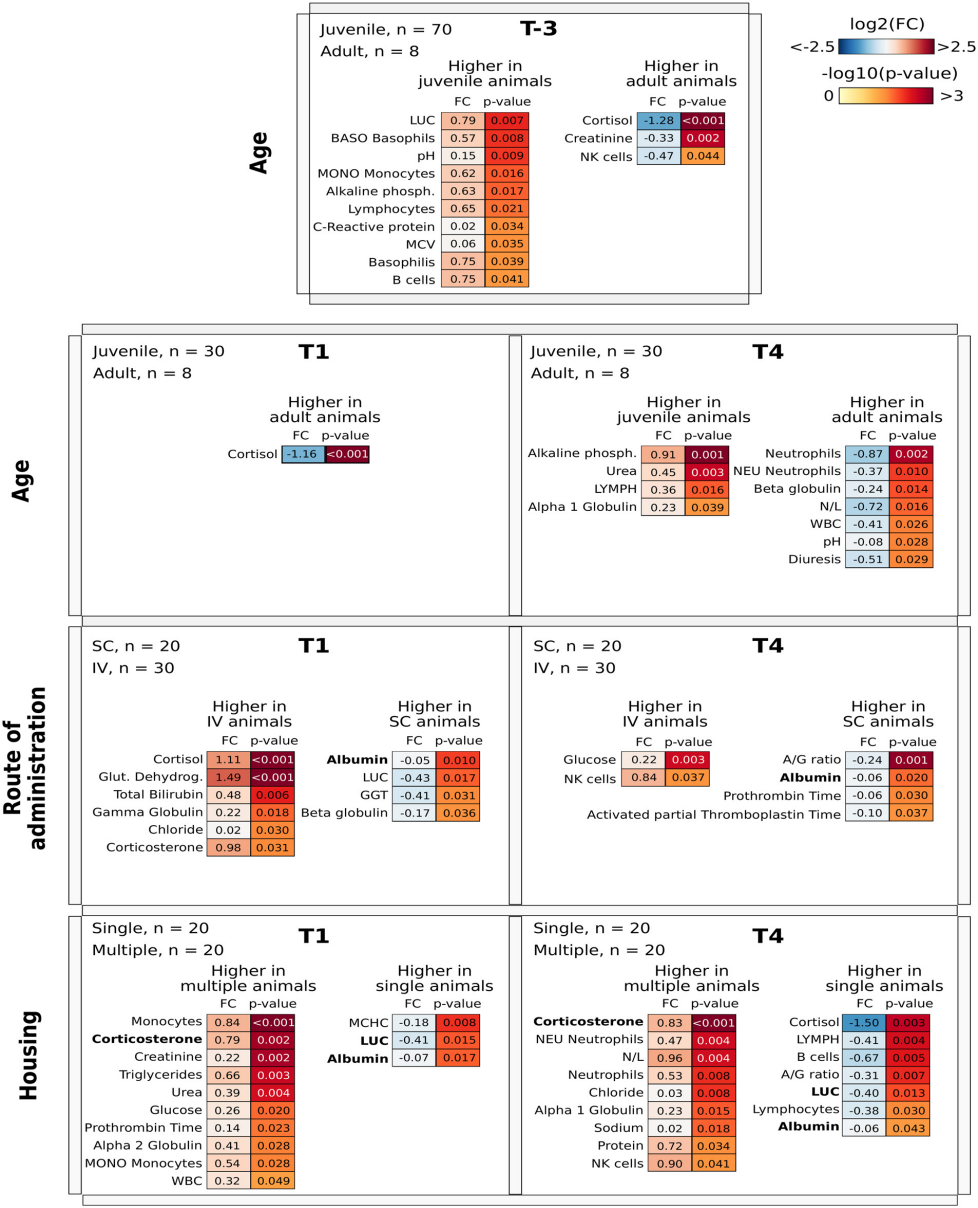
The four rows report the classes and the comparison between the categories considered. Each cell reports the time point considered, the number of samples used belonging the two categories and two heat map showing the log2 fold change (FC) of the measurements (left) and adjusted p-value computed using Wilcoxon Rank-Sum test (right). Note that the parameters are grouped into two heat maps based on their expression in the categories considered.

Firstly, for each comparison, the differences between the hematological and biochemical parameters measured were statistically evaluated using the rank-based nonparametric Wilcoxon test. Fig 3 shows a list of significant parameters organized based on the category and the time point. For the sake of clarity, in each comparison the parameters are grouped based on the category of the higher values. Concerning the administration route, albumin was found to be slightly higher in animals given subcutaneous injection than in those treated intravenously. Differences that emerged comparing housing were in corticosterone serum level and large unstained cells (LUC), with values being higher in multiple cage than in single cage animals for corticosterone and an opposite trend for LUC.

**Fig 4:**
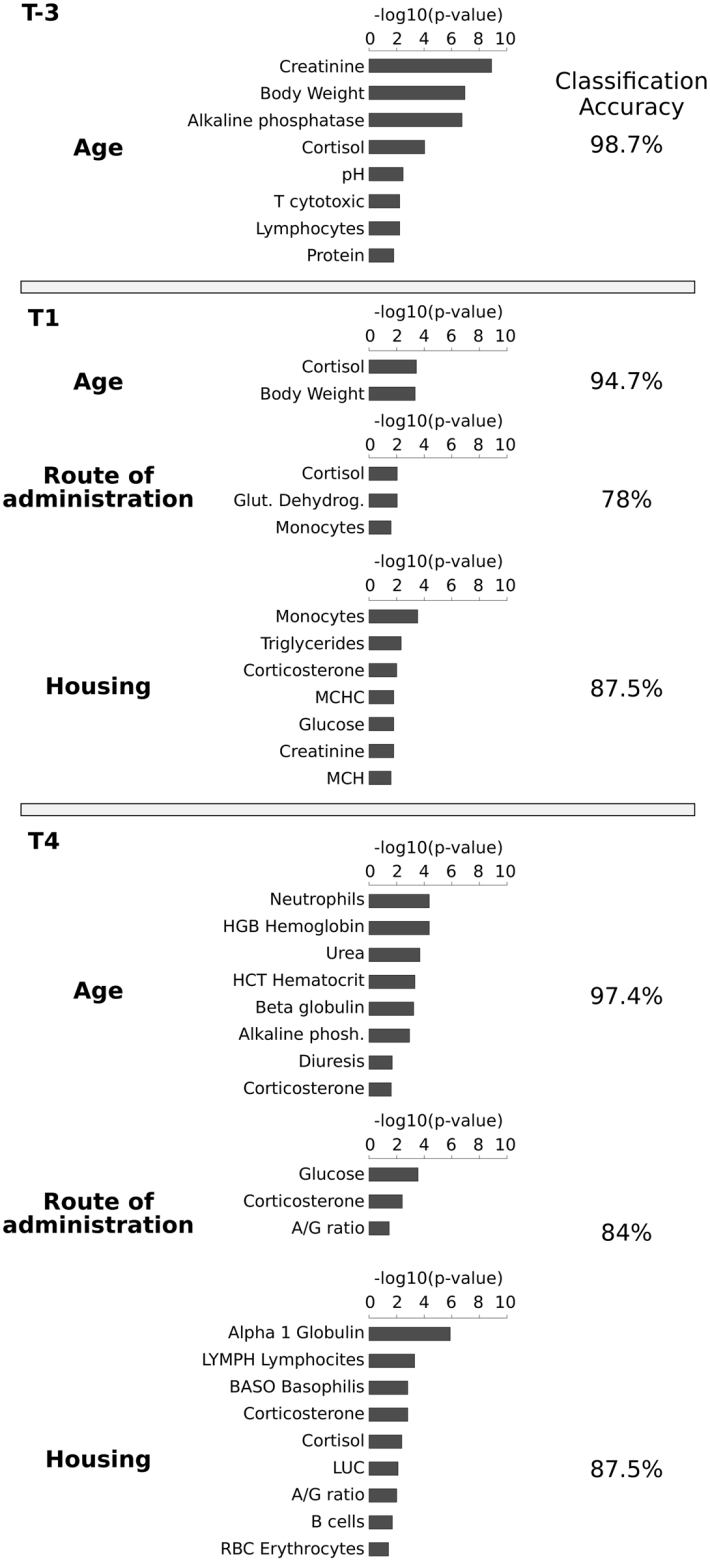
For each time point, it is showed a number of rows equal to the comparison between the categories considered. Each row shows the category considered (left), the bar plot reporting the p-value computed by the Chi-square test (center) for each parameter and the percentage of accuracy obtained by the classification algorithm (right). Only the results associated with a p-value < 0.05 were considered.

Secondly, a feature selection algorithm (*ChiSquareAttributeEvalfunction*, see Material and Methods) was used to evaluate the predictive properties of each parameter with respect to the class. For each category and time point, Fig 4 reports the list of attributes selected as being predictive for that category, namely *predictive parameters*. The Chi-square obtained for each parameter was transformed to p-values and showed as a bar plot (Fig 4 in the center). Low p-values should be interpreted as showing high worth of the parameter to determine the class identity of the animal.

Classification methods are able to classify an unknown sample into a predefined sample class. Fig 4 reports on the right the classification accuracy obtained for each class at each time point considering only the *predictive parameters*. It should be noted that the percentage of samples correctly classified was over the 78% for all categories. For the sake of completeness, three scenarios were compared: (i) all available parameters, (ii) without predictive parameters, and (iii) only predictive parameters. In five out of seven classification tests the set of predictive parameters has the highest percentage of correctly classified samples. The results are reported in the S2 Fig.

Note that corticosterone is the only parameter with a high discriminatory power at the T4 time point for each category (age, route of administration and housing). Moreover, corticosterone is also predictive for time point T1 in housing class. Although corticosterone was highlighted in both analyses, the information given is different: the Wilcoxon test identifies all parameters for which numerical values are significantly different between the categories of each class, while the features selection returns a list of *predictive parameters* able to discriminate between the categories of each class. In fact, the list of parameters obtained by the feature section algorithm can be used to provide the best classification performance for a predefined sample class. The N/L ratio did not show remarkable variations. As regards the FOB no additional, if any, stereotypes or other behavioral changes were observed. The statistical analysis of results did not highlight any significant differences.

### Time-course analysis

**Fig 5:**
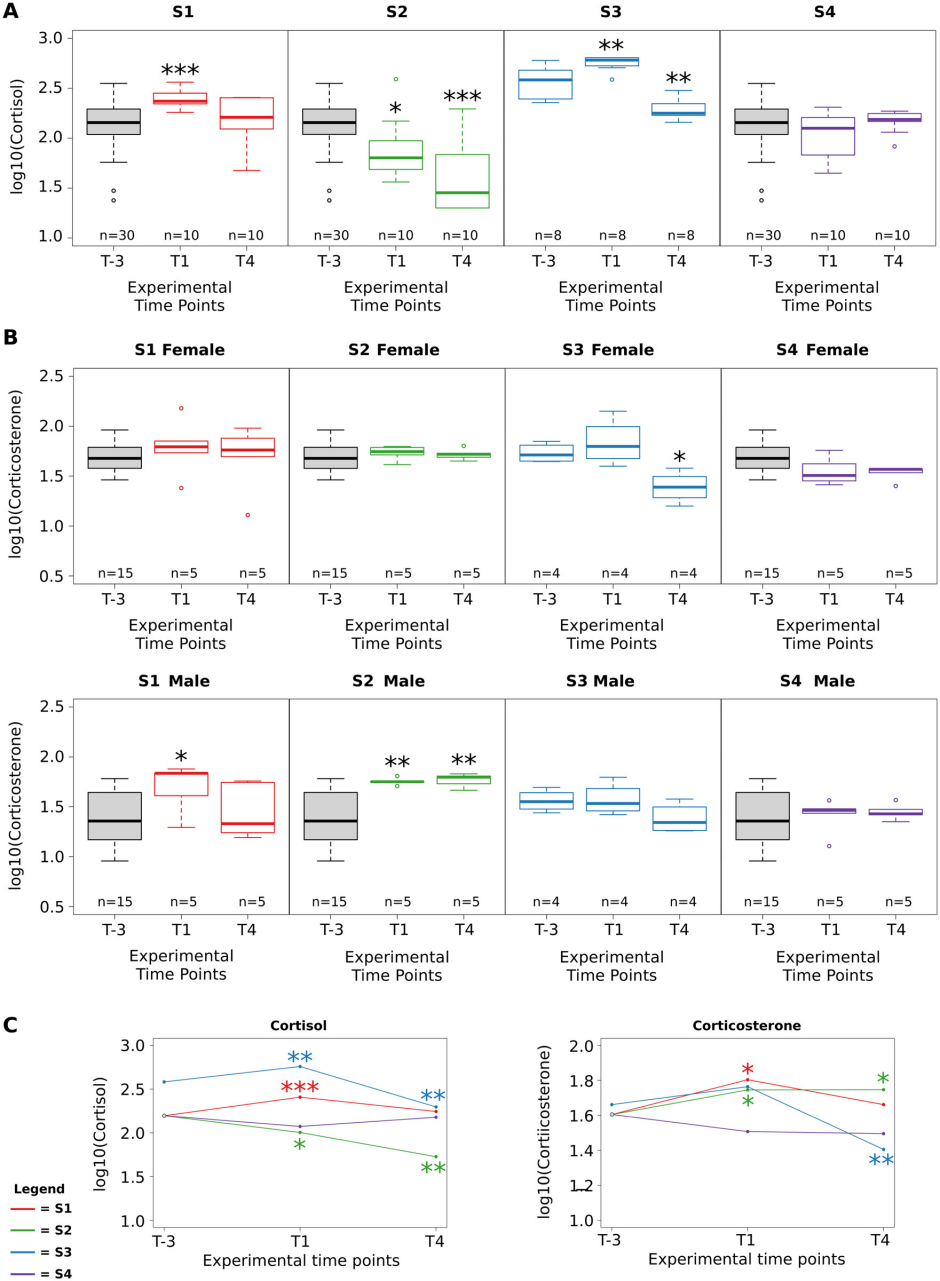
Box plots reporting cortisol and corticosteone values at each time point for each study, Panel A and B respectively. in Panel C Line plot reporting the average level of cortisol and corticosterone for each time point. The statistical significance of the comparison with respect to T-3 time point is reported. *** p-value < 0.0001, ** p-value < 0.01, * p-value < 0.05; Wilcoxon Rank-Sum test.

The goal of our analysis is to identify critical parameters able to indicate the experimental conditions that can cause stress in animals. The only parameter identified as being significant in several comparisons both from the Wilcoxon analysis and feature selection analysis is corticosterone level. For this reason, we explored the evolution trend of corticosterone compared with the dynamic trend of cortisol. In the time-course protocol, circulating levels of cortisol were also measured at each time point on a subset of animals. Fig 5A reports the cortisol level for each study at each time point. It should be noted that animals in Cohort 2 at time T-3 can be grouped only on the basis of their age since the administration route and housing could have no effect, i.e. the animals were not treated were all in single cages. To be consistent with the experimental design, the box plots at time T-3 were drawn considering all animals belonging to studies S1, S2 and S4. Note that the administration route had an impact at point T1 since only in studies S1 and S3 (where animals were treated by IV infusion) did the cortisol level increase with respect to the level at time T-3. Then, at T4 in both studies S1 and S3, the cortisol level decreased. Indeed, Fig 5C shows the temporal trend of cortisol (in a line plot) clearly evidencing the same dynamic of S1 and S3; however the S3 values are higher than the T1 values suggesting an effect caused by the age of the animals. Also a significant decrease in cortisol level can be observed at each time point in study S2, suggesting a possible relevant role of housing. Fig 5B reports the corticosterone level box plots for each study at each time point separately for male and female monkeys. Even though the number of animals when males and females are separated is very low, the trend in the corticosterone data observed in study S1 and S3 is similar to that observed in cortisol data. On the contrary, the corticosterone trend in study S2 is stable in female animals while it increases significantly in male animals.

## Discussion

The first aim of the study was to set up a baseline database from animals used for Toxicology studies at the RBM Merck research center. This database contains data from a total of 246 *cynomolgus monkeys* allowing the identification of parameters indicating experimental conditions that can cause stress in animals, in order to change, if necessary, working procedures. The animals were split into two cohorts used for different purposes. **Cohort 1** was composed of 238 juvenile animals, and was used to measure the effects of gender on hematological and biochemical measurements. Baseline samples were taken at the beginning of housing, between three and six weeks after the animals arrived at the research center.

We examined six published databases [5, 6, 16, 18, 19, 30] to compare our findings with recent literature data. These were chosen by first selecting papers on animals similar in terms of age, gender, body weight and housing conditions with respect to our **Cohort 1**. Below we discuss in detail the parameters associated with a high biological relevance.

The mean body weight measured in our animals was 3.1 Kg in males and 2.8 Kg in females. The difference was statistically significant, thus confirming the sexual dimorphism with respect to size already described by other authors [6, 19]. Red blood cell counts and related parameters hemoglobin and hematocrit were higher in males than in females, although the gender-related difference was very slight. No significant gender-dependent variations were found in other RBC-related parameters such as MCV, MCH and MCHC. RBC counts in male monkeys are known to exceed those in females after sexual maturity, occurring in general at about three to four years of age [31]. This finding may be related to the onset of the menstrual cycle in females, which is reported to begin at approximately 2.5 years of age. Neutrophil counts (both absolute and relative) were significantly increased in females versus males, whilst an opposite trend was observed for absolute and relative lymphocyte counts. This finding resulted in a significant increase in N/L ratio in female versus male animals. A significant difference was also observed for absolute and relative monocyte counts, with values in males exceeding those in females. However, due to the low absolute number of these cells, the difference was considered of minor biological relevance. As concerns white blood cells, the standard deviation of these parameters is similar to those reported in other databases, see S4 Table for details.

High inter-individual variability was reported for absolute values of all white blood cell subpopulations, and therefore any comparison between different databases should be made with caution. When considering relative counts, however, there was a general trend toward higher values of neutrophils in females and lymphocytes in males. This picture may be influenced by the age of the animals included in the databases, or by possible stress-inducing conditions, as may be the case of handling during blood sampling, resulting in the so-called *alarm reaction* that is characterized by hemoconcentration, lymphocytosis and neutrophilia [16, 19]. Coagulation parameters PT and APTT were similar in male and female animals.

Alkaline phosphatase serum levels were significantly higher in males than in females. Although in routine assays, the total AP measured includes liver, bone, kidney and intestinal isoenzymes [32], AP is commonly used as a biochemical marker of bone formation and osteoblast activity and consequently of skeletal growth velocity [6]. In general, AP values decline with increasing age, although with high inter-individual variability [5]. In a work by Perretta et al., AP levels were found to be higher in females than in males up to 36 months of age, whilst an opposite trend was seen in animals aged 37 months or more [6]. In our analysis, gender-related difference in AP mainly occurred in animals ranging from three to four years old; that could reflect the higher bone turnover in male animals of this age.

In our database, serum values of AST and GGT were significantly higher in males than in females. AST is a non-specific cytosolic and mitochondrial enzyme contained in hepatocytes, muscle fibers and red blood cells. AST values may increase during liver and muscle damage as well as in the case of hemolysis. Our values are in the same range as those observed in other papers [5, 6, 16, 18, 30]. No consistent gender-dependent difference was found among the various groups. Overall, although the gender-related difference was statistically significant, it was very mild and considered devoid of biological relevance. GGT is an enzyme associated with cell membranes, which is found with highest activity in the biliary, pancreatic and renal tubular epithelia. There is also evidence that cellular GGT plays an important role in antioxidant defense systems [33].

In monkeys, GGT is considered a more reliable marker of biliary effects than AP [34]. A decline in GGT levels with increasing age has also been reported [6]. In general, GGT values measured are lower than in other databases [5, 18]. However, the biological significance of the gender-related difference, as well as of the low values found in our study, are unclear.

Our analysis of serum protein electrophoresis showed a higher albumin fraction in males than in females and an opposite trend for globulin fraction, particularly for gamma-globulin. This finding resulted in a higher albumin/globulin ratio in males than in females. Total protein content showed no gender-related differences. These results are in agreement with those reported by Schuurman et al. [18], although there is no a clear explanation.

**Cohort 2** is composed of 78 animals (39 males and 39 females) that were included in the time-course protocol. In this analysis, data from male and female animals were considered together, since variations noted in the previous analysis were slight and values overall fell within the ranges of values described in all six databases examined. In addition to the standard clinical pathology examinations, body weight, FOB and urine analysis were determined at all time points in all animals. On the contrary, circulating levels of cortisol and corticosterone were measured only on a subset of animals, see Fig 5 for more details. We used statistical approaches (Wilcoxon test) and a machine learning approach (ChiSquare Attribute Evaluation) associated to a classification algorithm to achieve a panel of parameters potentially useful in assessing animal welfare. It should be pointed out that the different experimental conditions examined did not have any clinically visible influence on the animals’ behaviour.

Moreover, since circulating cortisol levels are known to increase in response to various kinds of stress, we verified whether there were other parameters correlated to the cortisol trend. In our data, a general trend toward a decrease in circulating cortisol levels with repeated treatment (T4 versus T1 and T-3) was observed in 3 out of 4 studies examined. The only exception was study S4, in which cortisol values were stable throughout the observation period. A gradual habituation to captive conditions is a known phenomenon described in experimental, potentially stress-inducing situations [20, 21, 24]. The decreases in cortisol values observed in our experiments could represent the possible effects of animals’ adaptation to chairing procedures or other experimental conditions. The significant decrease in cortisol levels at the time points T1 and T4 in the study S2 (in comparison with baseline levels and the other studies) may suggest a possible role of multiple caging in reducing the overall stress conditions of the animals. The temporal trend of cortisol levels showed the same dynamic in studies S1 and S3, both conducted by repeated intravenous treatment. However, the values at T1 appeared to be higher in study S3 (adult animals) than in S1 (juvenile animals), suggesting a possible effect due to the age of animals. Although our data do not allow a clear explanation of this effect, it could be supposed that the repeated manipulation of adult animals, in terms of capture and blood collection, may be more difficult and stressful due to their larger size. The lack of variations in cortisol levels in study S4 could indicate a lower impact of subcutaneous treatment in comparison with other routes of administration. Moreover, an inverse relationship with cortisol profile over time was found for serum alpha1 Globulin levels and partially for B-lymphocyte counts specially considering the study S2, see S3 Fig. This finding could be related to the immune-suppressive effect of cortisol [22, 24].

It should be noted that corticosterone is one of the most significant parameters in the Wilcoxon analysis and it is also the only parameter associated with high predictive power in four different comparisons namely, T1 and T4 both for routes of administration and housing. Circulating levels of corticosterone reflected the profile of cortisol. In rodents, corticosterone is the main glucocorticoid hormone. It is released by the adrenal cortex, and is the end product of the hypotalamic-pituitary-adrenal axis. Circulating corticosterone is known to increase above baseline levels in response to various stressors, such as extreme temperatures, increased physical activity or injury [35, 36]. Little is known about the role of circlulating corticosterone in non-human primates [37, 38], and therefore further studies will be necessary to better assess this parameter over time. We therefore cannot exclude or confirm a crucial role of circulating corticosterone in the assessment of animal stress.

Overall, no definitive conclusions can be drawn on the effect of the experimental conditions investigated in respect to conditions of acute stress. However, the fact that some statistically significant differences may be found already in cortisol baseline values may give useful indications on the putative parameters of stressful conditions to be assessed in future ad hoc studies, in which other parameters from different matrices, such as fecal and urinary cortisol [37, 39] measured with an appropriate sampling schedule, could be also considered. The use of a predictive methodology helps to define a panel of potential parameters able to assess animal welfare as demonstrated in the results of the classification methodology.

## Conclusion

In conclusion, we focused our analysis on two cohorts of *Macaca fascicularis* in order to to identify parameters indicating experimental conditions that can cause stress in animals. From **Cohort 1** the clinical pathology data may serve as background data for hematology, coagulation and clinical chemistry parameters of purpose-bred juvenile cynomolgus monkeys. Moreover, the time-course analysis on **Cohort 2** suggested that circulating cortisol levels may be used as a reliable marker to investigate animal stress under the common experimental conditions applied in research laboratories, whilst neutrophil/lymphocyte ratio as well as behavioral observations such as FOB do not appear to be significantly influenced by potentially stressing conditions.

## Supporting Information

S1 Fig List of the hematological and biochemical measurements tested between female and male animals of the Cohort 1. Each measurement corresponds to four columns: the average value and the standard deviation for females (i) and males (ii), (iii) log2 Fold Change (FC) of the measurements computed as females versus males (both represented as heat maps), and (iv) the p-values computed by Wilcoxon Rank-Sum test.
(PDF)

S2 Fig Bar plot of the percentage of correctly classified samples computed for each class grouped by time point. The first bar corresponds to the use of the classification algorithms given by all parameters, the second without the *predictive parameters*, and the last column the algorithm considers only *predictive parameters*.
(PDF)

S3 Fig Line plots reporting for each time point the average level of B-cells and alpha1 Globulin parameters. Dashed lines represent attributes lacking data for T1 time point. *** p-value < 0.0001, ** p-value < 0.01, * p-value < 0.05; Wilcoxon Rank-Sum test.
(PDF)

S1 Table All biochemical measurements and hematological values are listed for each sample of the Cohort 1. (XLS)

S2 Table The experiments, time points, sex, all biochemical measurements and hematological values are reported for each sample of the Cohort 2. (XLS)

S3 Table The number of animals in which a given parameter was measured at each time point is reported for each study. (XLS)

S4 Table The mean and standard deviation values of WBC, NEUT and LYMPH collected from 13 papers. (XLS)
