# Supplementary material for: Biologic Data of Cynomolgus Monkeys Maintained under Laboratory Conditions
Source: PLoS One. 2016 Jun 9;11(6):e0157003. doi: 10.1371/journal.pone.0157003 (PMC4900550; doi:10.1371/journal.pone.0157003)
Supplement: S1 Fig — Each measurement corresponds to four columns: the average value and the standard deviation for females (i) and males (ii), (iii) log2 Fold Change (FC) of the measurements computed as females versus males (both represented as heat maps), and (iv) the p-values computed by Wilcoxon Rank-Sum test. (PDF) [file pone.0157003.s001.pdf]

	Female (n=119) Avg ± SD	Male (n=119) Avg ± SD	FC	p-value
**Alkaline phosphatase (U/L)**	447.88 ± 144.02	647.07 ± 203.93	-0.53	< 0.001
**Gamma Globulin (%)**	17.39 ± 2.98	15.04 ± 2.76	0.21	< 0.001
**GGT (U/L)**	65.71 ± 22.18	83.31 ± 26.30	-0.34	< 0.001
**Chloride (mmol/L)**	109.13 ± 2.79	107.12 ± 2.63	0.03	< 0.001
**Inorganic Phosphorus (mmol/L)**	2.42 ± 1.30	2.73 ± 1.40	-0.17	< 0.001
**A/G ratio**	1.10 ± 0.14	1.23 ± 0.87	-0.14	< 0.001
**Albumin (%)**	51.53 ± 3.01	53.26 ± 3.25	-0.05	< 0.001
**Potassium (mmol/L)**	5.38 ± 0.76	5.66 ± 0.76	-0.07	< 0.01
**AST (U/L)**	40.73 ± 16.19	42.62 ± 11.75	-0.07	< 0.01
Total Bilirubin (µmol/L)	4.82 ± 1.78	4.49 ± 1.48	0.10	0.10
Alpha 1 Globulin (%)	5.03 ± 0.74	4.95 ± 0.78	0.02	0.13
Beta globulin (%)	18.09 ± 2.97	18.81 ± 3.31	-0.06	0.14
SGPT/ALT (U/L)	51.19 ± 23.61	48.58 ± 26.64	0.08	0.16
C-Reactive protein (mg/L)	1.32 ± 1.63	1.38 ± 1.44	-0.06	0.26
Creatinine (µmol/L)	75.66 ± 8.35	76.10 ± 10.98	-0.01	0.46
Sodium (mmol/L)	150.71 ± 3.29	150.53 ± 3.63	0.00	0.50
Glutamate Dehydrogenase (U/L)	29.29 ± 22.54	26.71 ± 16.90	0.13	0.54
Urea (mmol/L)	7.59 ± 1.89	7.60 ± 1.78	0.00	0.63
Triglycerides (mmol/L)	0.56 ± 0.24	0.54 ± 0.22	0.05	0.76
Glucose (mmol/L)	3.79 ± 0.87	3.75 ± 0.78	0.02	0.79
Alpha 2 Globulin (%)	8.02 ± 2.41	7.92 ± 2.44	0.02	0.79
Total protein (g/L)	82.57 ± 4.87	75.37 ± 5.01	0.13	0.82
Calcium (mmol/L)	3.58 ± 2.66	3.54 ± 2.64	0.02	0.86
Total Cholesterol (mmol/L)	4.04 ± 0.81	4.08 ± 0.90	-0.01	0.92

	Female (n=118) Avg ± SD	Male (n=118) Avg ± SD	FC	p-value
**N/L**	1.50 ± 1.09	0.78 ± 0.52	0.94	< 0.001
**NEU Neutrophils (%)**	50.92 ± 14.74	37.98 ± 13.77	0.42	< 0.001
**LYMPH Lymphocites (%)**	44.39 ± 14.04	56.66 ± 13.33	-0.35	< 0.001
**Neutrophils (10E3 cells/mcL)**	7.05 ± 3.63	4.95 ± 2.28	0.51	< 0.001
**Lymphocytes (10E3 cells/mcL)**	5.71 ± 2.32	7.55 ± 3.40	-0.40	< 0.001
**HGB Hemoglobin(g/dL)**	13.71 ± 0.94	14.02 ± 1.44	-0.03	< 0.001
**MONO Monocytes (%)**	2.91 ± 1.09	3.46 ± 1.20	-0.25	< 0.001
**Monocytes (10E3 cells/mcL)**	0.37 ± 0.18	0.45 ± 0.21	-0.28	< 0.01
**Large Unstained Cells (%)**	0.74 ± 0.35	0.83 ± 0.38	-0.17	< 0.05
**BASO Basophilis (%)**	0.37 ± 0.10	0.41 ± 0.13	-0.15	< 0.05
**HCT Hematocrit(%)**	46.34 ± 3.78	47.27 ± 3.52	-0.03	< 0.05
**RBC Erythrocytes (10E6 cells/mcL)**	5.93 ± 0.45	6.06 ± 0.41	-0.03	< 0.05
**MCHC (g/dL)**	27.88 ± 3.26	28.31 ± 3.15	-0.02	< 0.05
Basophilis (10 E3 cells/mcL)	0.05 ± 0.02	0.06 ± 0.04	-0.26	0.08
PLT Plateles (10E3 cells/mcL)	414.56 ± 82.63	407.52 ± 143.01	0.02	0.10
RET Reticulocytes (10E9 cells/L)	56.37 ± 19.82	63.24 ± 33.14	-0.17	0.28
Prothrombin Time (sec)	12.59 ± 1.19	12.39 ± 0.84	0.02	0.30
Reticulocytes (%)	0.95 ± 0.34	1.01 ± 0.62	-0.09	0.34
EOS Eosinophilis (%)	0.66 ± 0.65	0.63 ± 0.50	0.07	0.44
Act. partial Thromboplastin Time (sec)	17.85 ± 1.96	17.66 ± 1.95	0.02	0.49
MCH (pg)	24.87 ± 2.97	25.04 ± 2.99	-0.01	0.74
Eosinophilis (10E3 cells/mcL)	0.09 ± 0.10	0.09 ± 0.10	0.00	0.84
MCV Means Corpuscular Volume (fL)	78.19 ± 3.90	78.09 ± 3.78	0.00	0.84
WBC Leukocytes (10E3 cells/mcL)	12.97 ± 3.40	13.21 ± 4.12	-0.03	0.92

-log10(p-value)

0 >10

log2(FC)

-1 1
