# Supplementary figures and images for: Biologic Data of Cynomolgus Monkeys Maintained under Laboratory Conditions
Source: PLoS One. 2016 Jun 9;11(6):e0157003. doi: 10.1371/journal.pone.0157003 (PMC4900550; doi:10.1371/journal.pone.0157003)

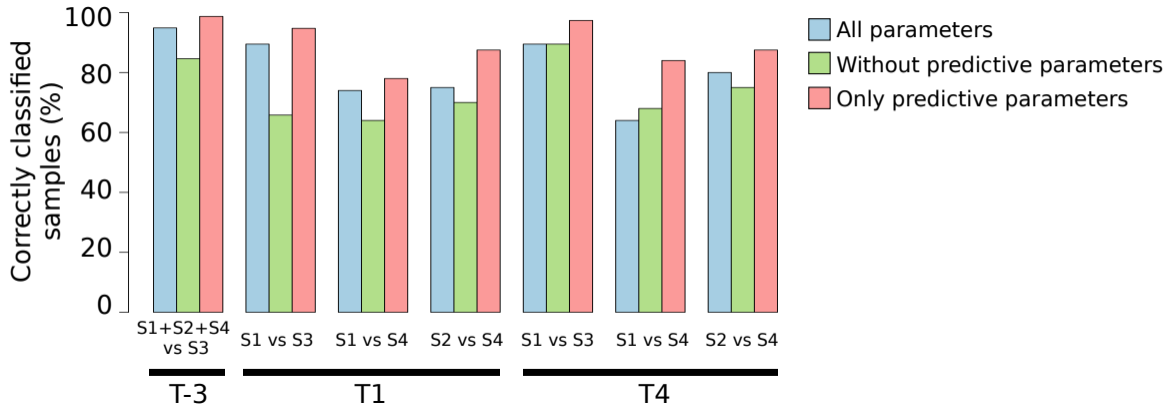

Supplement: S2 Fig — The first bar corresponds to the use of the classification algorithms given by all parameters, the second without the predictive parameters, and the last column the algorithm considers only predictive parameters. (PDF) [file pone.0157003.s002.pdf]

**B cells (%)**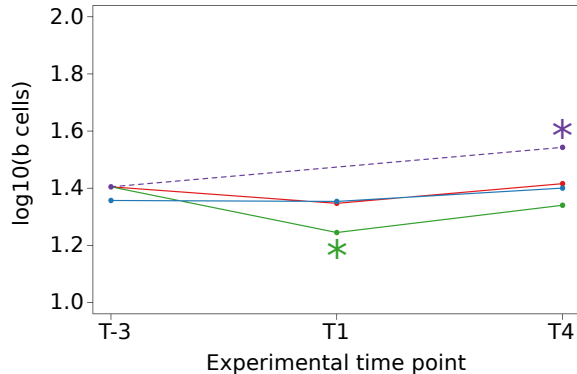

|       | B cells (%) - n° samples |    |    |
|-------|--------------------------|----|----|
| Study | T-3                      | T1 | T4 |
| S1    | 10                       | 10 | 10 |
| S2    | 9                        | 10 | 10 |
| S3    | 8                        | 8  | 8  |
| S4    | 10                       | 0  | 10 |

**Alpha 1 Globulin (%)**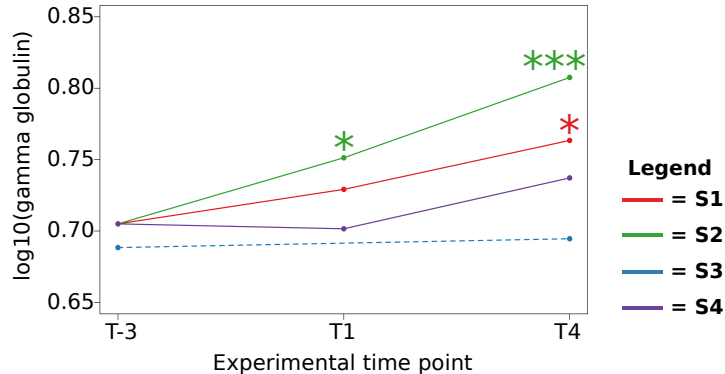

|       | Alpha 1 Globulin (%) - n° samples |    |    |
|-------|-----------------------------------|----|----|
| Study | T-3                               | T1 | T4 |
| S1    | 30                                | 30 | 20 |
| S2    | 20                                | 20 | 20 |
| S3    | 8                                 | 0  | 8  |
| S4    | 20                                | 10 | 20 |

Supplement: S3 Fig — Dashed lines represent attributes lacking data for T1 time point. *** p-value < 0.0001, ** p-value < 0.01, * p-value < 0.05; Wilcoxon Rank-Sum test. (PDF) [file pone.0157003.s003.pdf]
